# Growth-Based Decision-Making in Congenital Scoliosis with Multiple Vertebral Anomalies

**DOI:** 10.3390/jcm15062198

**Published:** 2026-03-13

**Authors:** Seidali Abdaliyev, Daniyar Yestay, Dina Saginova, Alexander Chsherbina, Daulet Baitov, Serik Serikov

**Affiliations:** 1 National Scientific Center for Traumatology and Orthopedics Named After Academician N.D. Batpenov, Astana 010000, Kazakhstan; 2 The Institute of Life Sciences, Karaganda Medical University, Karaganda 100000, Kazakhstan

**Keywords:** congenital scoliosis, vertebral growth potential, growth modulation, mechanobiology, timing of intervention

## Abstract

**Background**: Congenital scoliosis (CS) associated with multiple vertebral anomalies (MVAs) represents a biologically dynamic deformity in which cumulative segmental asymmetry, residual growth potential, and mechanobiological modulation interact to drive progression. Unlike isolated congenital lesions, MVAs exhibit growth-dependent and configuration-specific behavior, complicating risk stratification and timing of intervention. Despite extensive literature on congenital deformities, an integrated growth-oriented decision framework for this subgroup remains lacking. **Methods**: This narrative review synthesizes embryological, biomechanical, and clinical evidence related to vertebral growth potential, anomaly configuration, progression patterns, and age-dependent treatment strategies in CS with MVAs. A structured literature search of major databases was performed, and findings were analyzed thematically to propose a biologically grounded growth-based decision framework. **Results**: Across the literature, three interdependent determinants of progression consistently emerge: anomaly configuration, residual segmental growth capacity, and mechanobiological amplification during growth. High-risk configurations—particularly mixed formation–segmentation defects and fully segmented hemivertebrae with contralateral growth arrest—demonstrate rapid and often non-linear progression. Thoracic involvement further modifies clinical urgency due to its impact on pulmonary development. Integration of developmental biology and mechanobiological principles supports a structured, growth-informed approach to surveillance and intervention timing. **Conclusions**: MVAs should be conceptualized as dynamic growth systems rather than static structural defects. A shift from angle-driven to growth-informed decision-making may enhance early identification of high-risk patterns while minimizing unnecessary premature fusion in lower-risk cases. Adoption of a structured growth-based framework provides a biologically coherent foundation for individualized management and long-term optimization of spinal and thoracic development.

## 1. Introduction

Congenital scoliosis (CS) arises from abnormal vertebral development in utero and is characterized by structural malformations such as failures of formation, failures of segmentation, or mixed defects [1,2,3]. Typical anomalies—including hemivertebrae, unilateral unsegmented bars, and complex combined malformations—introduce intrinsic asymmetry into spinal growth and predispose to progressive curvature [1,2,3,4]. Among affected patients, those with multiple vertebral anomalies (MVAs) represent a particularly complex and clinically challenging subgroup. In contrast to isolated defects, deformity progression in MVAs is driven by the cumulative and interacting growth behavior of multiple anomalous segments, resulting in dynamic three-dimensional imbalance during periods of accelerated spinal growth.

Specific anomaly configurations exhibit distinct progression patterns. Adjacent ipsilateral hemivertebrae may generate sharp, short-segment curves, whereas alternating anomalies can produce double or S-shaped deformities. Mixed configurations particularly a hemivertebra opposite a contralateral unsegmented bar are associated with the most severe and rapidly progressive curves. In such cases, curve behavior reflects not only structural abnormality but also residual vertebral growth potential and mechanobiological modulation [3,4,5,6].

The clinical course of congenital scoliosis is heterogeneous. While some deformities remain relatively stable, others progress relentlessly during growth and may lead to severe truncal deformity and thoracic insufficiency syndrome, compromising pulmonary development and cardiopulmonary function [5,7,8]. Conversely, extensive early spinal fusion may impair spinal and thoracic growth and contribute to long-term morbidity [9,10]. Management therefore requires a careful balance between preventing irreversible deformity and preserving physiological growth.

Although prior reviews have addressed classification systems and surgical techniques, there remains no integrated framework specifically focused on growth-based decision-making in patients with multiple vertebral anomalies. In this subgroup, conventional algorithms derived from isolated anomalies may be insufficient, as cumulative asymmetric growth, mechanobiological principles, and age-dependent growth velocity collectively determine deformity trajectory.

In this narrative review, we synthesize current evidence on vertebral growth potential, mechanobiological modulation, and age-dependent progression patterns in congenital scoliosis with multiple vertebral anomalies. Our aim is to propose a structured, biologically informed framework for risk stratification and timing of intervention, thereby supporting individualized clinical decision-making and optimization of long-term spinal and thoracic development.

## 2. Materials and Methods

### 2.1. Study Design

This study was conducted as a narrative review using a structured literature search strategy. Given the heterogeneity of study designs, patient populations, and reported outcomes in congenital scoliosis with multiple vertebral anomalies, quantitative synthesis was not considered appropriate. Instead, a narrative synthesis approach was used to integrate embryological, biomechanical, and clinical evidence relevant to vertebral growth and deformity progression.

### 2.2. Search Strategy

**Figure 1:**
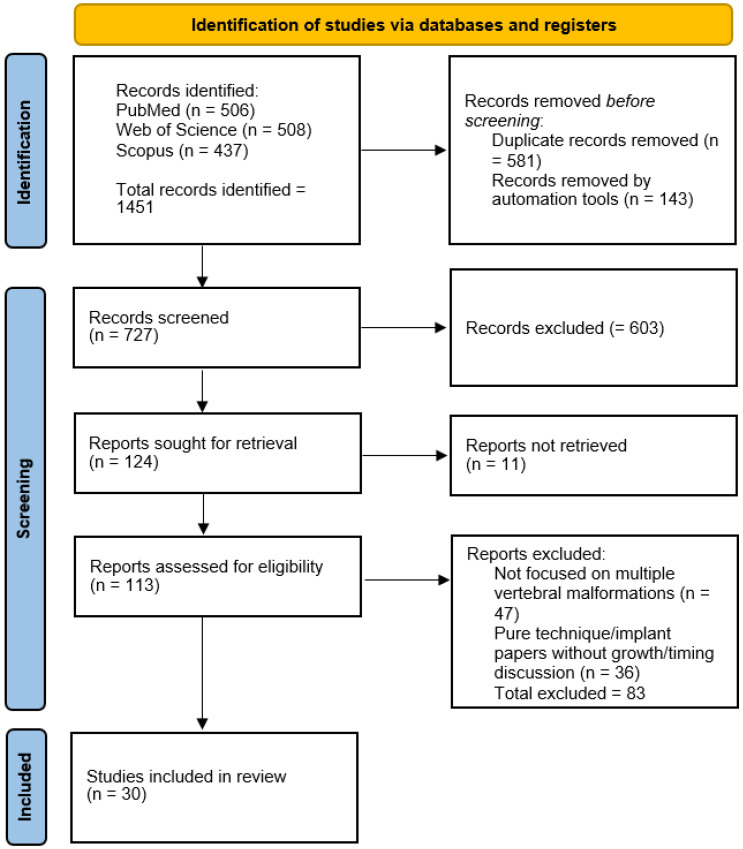
PRISMA 2020 flow diagram illustrating the study selection process.

A structured literature search was performed in PubMed, Web of Science, and Scopus databases. The primary search covered studies published between January 2000 and March 2025. Only English-language publications were considered. Search terms included combinations of “congenital scoliosis”, “multiple vertebral anomalies”, “hemivertebra”, “vertebral growth”, “mechanobiology”, “thoracic insufficiency”, “growth modulation”, and “timing of intervention”. Automated database filters were applied for language and publication year prior to screening. Study selection is reported using a PRISMA 2020 flow diagram to enhance transparency, although the synthesis is narrative (Figure 1).

### 2.3. Study Selection

After removal of duplicate records and automated exclusions, titles and abstracts were screened for relevance. Studies were considered eligible if they addressed congenital vertebral malformations with relevance to vertebral growth potential, deformity progression, mechanobiological mechanisms, or timing of intervention. Exclusion criteria included studies not focused on congenital vertebral malformations, unrelated spinal pathology, purely technical surgical reports without analysis of growth or progression, duplicate or overlapping patient cohorts, and studies lacking sufficient methodological detail. Full-text assessment was performed for potentially eligible studies. In addition to the structured database search, seminal studies relevant to vertebral growth physiology, mechanobiological principles, and early characterization of congenital vertebral malformations were identified through manual reference screening.

### 2.4. Data Synthesis

Given the substantial heterogeneity in study designs and outcome measures, findings were synthesized narratively. Particular emphasis was placed on vertebral growth behavior, anomaly configuration, progression patterns, and age-dependent clinical implications. The synthesized evidence informed the development of a growth-based conceptual framework intended to support clinical risk stratification and timing of intervention in patients with multiple vertebral anomalies.

## 3. Results

Across the included literature, deformity behavior in congenital scoliosis with multiple vertebral anomalies is described within three interrelated thematic domains: (1) embryologic anomaly configuration, (2) residual growth potential of affected segments, and (3) mechanobiological modulation during spinal growth. The following sections synthesize embryological, biomechanical, and clinical evidence within these domains.

### 3.1. Embryogenesis of the Vertebrae and Congenital Anomalies

The vertebral column develops early in embryogenesis through notochord patterning beginning in the 3rd week of gestation, followed by differentiation of paraxial mesoderm into somites during weeks 4–5 [1,2,7,11,12]. The sclerotome component of each somite gives rise to the vertebrae and ribs and undergoes re-segmentation during the 6th week, establishing vertebral primordia and intervertebral structures [2,7,13]. Disruption of these processes results in congenital vertebral malformations (CVMs). Failures of formation lead to anomalies such as hemivertebrae or wedge vertebrae, whereas failures of segmentation result in bony bridges or block vertebrae. Mixed defects are frequent; a unilateral unsegmented bar with a contralateral hemivertebra represents a classic configuration associated with high progression potential [1,7].

The timing of disturbances, typically between weeks 4 and 6 of gestation, has been described as a developmental explanation for associations with anomalies in other organ systems developing concurrently, including syndromic contexts such as VACTERL association and Jarcho–Levin syndrome [2,7,11]. Genetic studies have identified mutations in key regulators of somitogenesis and segmentation, including MESP2, DSTYK, HES7, and TBX6, particularly in disorders such as spondylocostal dysostosis [1,13,14,15,16]. However, most cases of congenital scoliosis appear multifactorial, arising from polygenic susceptibility combined with environmental influences such as maternal diabetes, hypoxia, or teratogenic exposure (e.g., retinoic acid) [1,2,7]. Whole-exome sequencing identifies likely pathogenic variants in a minority of patients (approximately 18%), consistent with the complexity of vertebral development [1].

### 3.2. Postnatal Vertebral Growth and Physiology

Normal spinal growth provides the biological substrate through which congenital anomalies translate into deformity progression. Vertebral growth occurs through vertebral endplates, ring apophyses, and intervertebral discs. Spinal growth is age dependent: approximately 50% of trunk growth occurs within the first five years of life, followed by about 30% between ages five and puberty, and the remaining 20% during the adolescent growth spurt [17]. Dimeglio and colleagues reported that by age five, 80–90% of thoracic spinal canal growth is achieved, and by age ten approximately 80% of sitting height is attained [17]. Two acceleration periods early childhood and puberty have been described as clinically relevant growth windows in which progression may accelerate [16,17].

Growth potential of an anomalous vertebral segment depends on the presence and configuration of functional growth regions. A fully segmented hemivertebra, bounded by intervertebral discs above and below, retains two functional endplate physes and can contribute to asymmetric longitudinal growth. In contrast, semi-segmented hemivertebrae have reduced growth capacity, while non-segmented hemivertebrae and block vertebrae have limited effective vertical growth potential due to absent or fused growth regions [1,2,6]. Anatomical location further modulates clinical impact: thoracic anomalies have been described as more likely to affect thoracic cage development and pulmonary growth than lumbar anomalies [1,2,5,16]. According to the Hueter–Volkmann principle, compressive forces inhibit growth whereas tensile forces promote it; asymmetric loading across vertebral bodies and discs can therefore modulate growth rates and amplify pre-existing asymmetries introduced by congenital anomalies [18,19].

### 3.3. Patterns of Growth Potential in Multiple Vertebral Anomalies

Congenital scoliosis associated with multiple vertebral anomalies demonstrates heterogeneous progression patterns determined by the type, configuration, and location of malformations. Growth potential refers to the capacity of an anomalous spinal segment to continue growing asymmetrically and exacerbate deformity. Certain anomaly combinations are consistently associated with rapid progression, whereas others may remain relatively balanced or minimally progressive [20,21]. Longitudinal studies have shown that mixed anomalies combining a formation defect with a contralateral segmentation defect carry the highest risk of progression [20]. A fully segmented hemivertebra opposite an unsegmented bar has been reported to show rapid progression, commonly reaching 5–10° per year during growth [4,6,22]. Multiple consecutive fully segmented hemivertebrae on the same side generate steep, short-segment curves and demonstrate higher progression rates compared with isolated lesions in clinical series [12]. Other multiple-anomaly patterns may exhibit partial compensation; for example, hemivertebrae located on opposite sides of the spine and separated by intervening normal segments may produce balanced double curves, although kyphotic components are commonly described in multilevel congenital deformities. Anomaly location also modulates clinical presentation and compensatory capacity, including junctional regions such as cervicothoracic or lumbosacral levels [4,6].

Quantitative natural history data underscore the predominantly progressive nature of congenital scoliosis. McMaster and Ohtsuka reported that approximately 75% of congenital curves progressed over time, with the most rapid progression observed in unilateral unsegmented bars with contralateral hemivertebrae, often exceeding 5° per year [23]. Progression has been described as non-linear, with acceleration reported during early childhood and again during the pubertal growth spurt [12].

### 3.4. Vertebral Modulation and Mechanobiology

Progression of scoliosis in the growing spine is influenced by biomechanical forces acting on a biologically active growth system. Mechanobiology describes the interaction between mechanical loading and vertebral growth, whereby asymmetric forces across the spine modulate growth rates on opposite sides of vertebral bodies and intervertebral discs [1,6,19,24]. The Hueter–Volkmann principle provides a conceptual basis whereby compression inhibits and relative unloading promotes growth. Once curvature exists, differential loading can lead to progressive wedging of vertebral bodies and discs and contribute to a self-perpetuating “vicious cycle” [4,19,25,26,27]. Intervertebral discs contribute substantially to spinal height, and both vertebral body wedging and disc wedging have been described as contributors to progression; vertebral wedging is commonly emphasized in thoracic curves, while disc wedging may be more prominent in lumbar regions [28].

Experimental studies support the plausibility of mechanical growth modulation. Asymmetric loading applied to immature spines has been shown to produce wedging and scoliotic deformities even without intrinsic malformations [24,28,29]. Unilateral growth restraint across vertebral growth regions in animal models resulted in predictable curve formation with suppressed physeal activity on the restrained side, paralleling mechanisms exploited by hemiepiphysiodesis approaches [29,30].

### 3.5. Clinical Growth Modulation: Bracing, Casting, and Hemiepiphysiodesis

Mechanobiological principles have been translated into clinical strategies intended to influence growth while avoiding or delaying definitive fusion [24,27,28]. Although bracing is primarily associated with idiopathic scoliosis, selected studies describe serial casting or bracing in mild congenital scoliosis or very young patients to guide growth and slow progression, including combinations such as Risser casting and bracing and custom orthoses [1,8,18,31]. Wang et al. reported that bracing in congenital scoliosis can postpone surgical intervention by moderating progression during early growth phases [18]. These approaches have been described mainly as temporizing measures rather than definitive treatment [1,25].

Convex hemiepiphysiodesis (convex growth arrest) restrains convex-side growth while preserving concave-side growth. Modern techniques include instrumented hemiepiphysiodesis using screws and rods to provide initial correction while acting as a growth restraint [19,25]. Long-term follow-up studies have reported gradual improvement when hemiepiphysiodesis is performed early, particularly in very young patients with moderate curves (e.g., <35–40°) [32]. Outcomes are described as less predictable with increasing age or curve magnitude, and hemiepiphysiodesis alone may be insufficient in older children or rapidly progressive deformities.

Anterior vertebral body stapling or tethering has emerged primarily in idiopathic scoliosis; its role in congenital scoliosis remains limited due to structural bony anomalies, but isolated reports describe use in selected mild congenital or mixed deformities with remaining growth. These techniques are generally described as investigational in congenital scoliosis, with potential future applicability as technologies evolve [33,34,35].

### 3.6. Timing of Surgical and Growth-Preserving Interventions (Evidence Summary)

Clinical reports and reviews describe timing as influenced by remaining spinal growth, curve severity and progression rate, and thoracic/pulmonary considerations. Multiple authors have emphasized intervention before curves become large and rigid; thresholds such as 40–50° in young children are frequently cited in the literature [16,20,36]. Burnei et al. proposed a Cobb angle of 40° as a practical threshold in clinical decision pathways, differentiating close observation below this value from earlier surgery when progression reaches or exceeds 40° in early childhood [12]. Hemivertebra resection with short-segment fusion is commonly described in toddlers with sharp progressive curves, with reported substantial correction and short fused segments [1]. Several authors have reported superior deformity correction and no adverse effect on spinal growth or pulmonary development when surgery was performed before the age of six compared with later intervention [21]. Qian et al. compared posterior hemivertebra resection outcomes in children operated at ≤5 versus >5 years and reported comparable radiographic correction, but higher unplanned reoperation rates in younger patients; the authors suggested that in selected situations, delaying surgery until early childhood may reduce complication risk [22].

For long-segment deformities or multiple anomalies, growth-friendly strategies have been developed to preserve growth, including growing rods and vertical expandable prosthetic titanium ribs (VEPTR) [12,16,19]. Dual growing rod constructs have been reported to control curves while permitting continued spinal growth, averaging approximately 1 cm of spinal length gain per year in series reports [1,19]. Complications such as rod breakage, infection, and autofusion are reported concerns, prompting development of magnetically controlled rods and growth-guidance systems such as Shilla-type techniques [1,16,19]. VEPTR was developed for thoracic insufficiency syndrome associated with congenital scoliosis and rib anomalies [16,34]; clinical studies report improved thoracic growth and lung volume in selected patients treated with VEPTR, with early use described when thoracic volume is critically compromised [19,20].

Definitive fusion is often described as delayed until later childhood or early adolescence; fusion performed after approximately 10–12 years of age has been reported to have a lower impact on final height and pulmonary capacity than fusion performed in infancy [2,12,21]. However, prolonged delay in the presence of uncontrolled progression may lead to rigid deformity and clinical sequelae, and timing is described as individualized based on deformity behavior and response to interim strategies.

### 3.7. Thoracic and Pulmonary Development Considerations

A defining concern in early-onset and congenital scoliosis is impact on thoracic and pulmonary development. Multiple vertebral anomalies may affect the rib cage directly (rib fusion or malformation) or indirectly via altered rib orientation from spinal deformity, contributing to thoracic insufficiency syndrome (TIS), defined as the inability of the chest to support normal respiration or lung growth [4,10,37,38].

Early childhood is described as a critical window for pulmonary development, with substantial alveolar multiplication and chest growth occurring within the first five years of life and continuing through puberty. Severe thoracic deformity during this period may result in asymmetric thoracic development, reduced hemithoracic volume, and lung hypoplasia, particularly on the concave side. Rib fusion in syndromic contexts such as Jarcho–Levin or spondylothoracic dysplasia has been described as further restricting chest wall expansion and exacerbating pulmonary compromise [4,10,38]. Contemporary reviews report that early-onset scoliosis can adversely affect pulmonary development, particularly when deformity and rib cage distortion are untreated [10,19]. Imaging and functional assessments described in clinical practice include three-dimensional CT to quantify hemithoracic volume and identify rib anomalies, and pulmonary function testing in older cooperative children [5].

## 4. Discussion

The synthesized literature supports a growth-dependent view of congenital scoliosis with multiple vertebral anomalies, in which deformity progression reflects dynamic interaction among anomaly configuration, residual growth potential, and mechanobiological amplification during growth. These determinants operate cumulatively rather than independently, helping explain why MVAs demonstrate heterogeneous but frequently progressive trajectories.

### 4.1. Clinical Interpretation of Growth Potential Patterns

From a clinical standpoint, recognition of growth potential patterns can inform risk stratification and timing of intervention. High-risk configurations—particularly thoracic mixed anomalies such as a hemivertebra opposite a contralateral unsegmented bar—have been reported to demonstrate rapid progression, often leading to severe deformity early in life if untreated [4,6,22,23]. Congenital vertebral malformations may also coexist with intraspinal anomalies such as tethered cord or diastematomyelia, which can influence surgical strategy and neurological risk [3,5,7,39]. Multiple consecutive fully segmented hemivertebrae on the same side similarly demonstrate high progression rates compared with isolated lesions [12]. Conversely, some alternating patterns may provide partial coronal compensation, although kyphotic components are common in multilevel congenital deformities and progression can still occur [12,23]. Given that natural history data suggest that the majority of congenital curves progress over time [23], and that progression may accelerate during early childhood and the pubertal growth spurt [12,17], longitudinal follow-up remains essential even in apparently lower-risk configurations.

### 4.2. Mechanobiology in Decision-Making

Mechanobiological principles provide a rationale for why the mechanical environment can amplify congenital asymmetry during growth and why growth modulation may have clinical leverage in selected cases [1,6,19,24]. Growth modulation strategies aim to alter asymmetric loading conditions consistent with Hueter–Volkmann-type principles, whereas fusion strategies must account for the risk of creating additional growth imbalance. Mechanobiology is also central to understanding crankshaft, in which continued anterior growth after isolated posterior fusion can drive progressive rotation and deformity in skeletally immature patients [17,19,24,28]. These considerations support aligning treatment selection with the presence of remaining growth and the availability of concave-side growth potential.

### 4.3. Growth-Based Risk Stratification and Timing Framework

**Figure 2:**
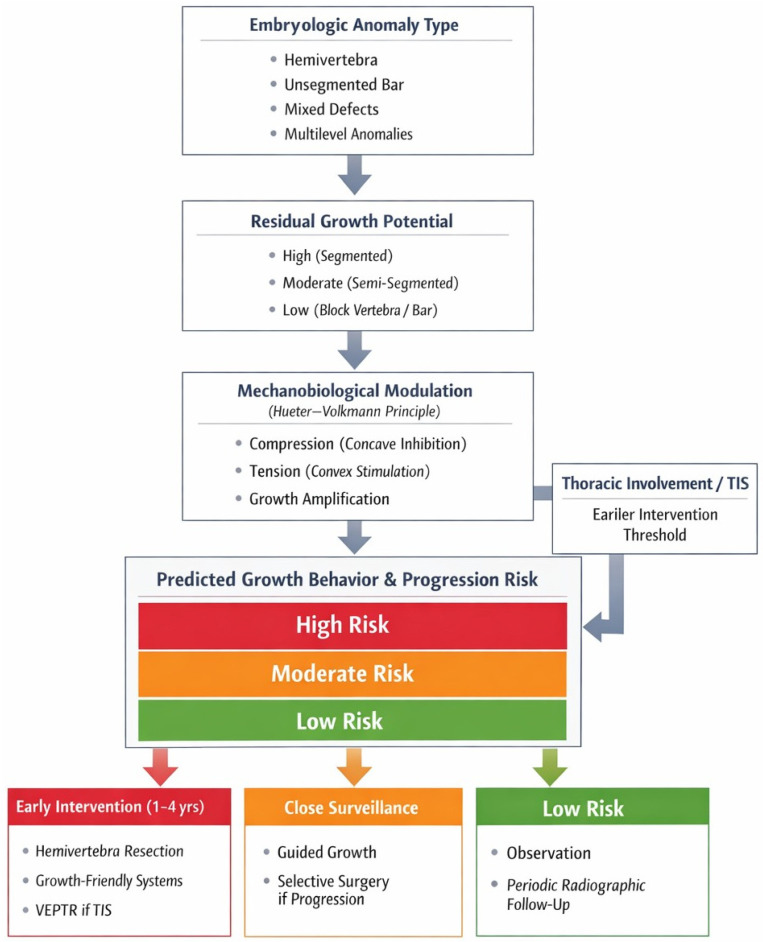
Conceptual Growth-Based Risk Stratification and Clinical Decision Framework for Congenital Scoliosis with Multiple Vertebral Anomalies.

Figure 2 illustrates the proposed Growth-Based Risk Stratification and Timing Framework for congenital scoliosis with multiple vertebral anomalies. The framework is built on four interrelated determinants of deformity behavior: (i) anomaly configuration and segmentation status, (ii) residual vertebral growth potential, (iii) mechanobiological amplification during growth, and (iv) thoracic and pulmonary constraints. Integration of these factors provides a biologically informed basis for risk stratification and clinical decision-making. Based on the synthesis of natural history and clinical outcome studies, common anomaly patterns can be stratified according to growth potential, progression risk, and timing or strategy pathways (Table 1).

### 4.4. Timing Windows and Strategy Selection (Evidence-Informed)

Evidence from clinical series suggests that timing decisions must be individualized according to progression rate, configuration-dependent risk, and age-related procedural trade-offs. Traditional paradigms emphasize earlier correction of progressive deformities to prevent the development of large, rigid curves requiring extensive fusion [16,20,36]. Thresholds around 40–50° in young children are frequently referenced [16,20,36], and Burnei et al. proposed a 40° threshold as a practical decision point in early childhood [12]. At the same time, comparative data indicate that very early surgery can be associated with higher unplanned reoperation rates, supporting delayed intervention into early childhood when progression is not rapid and deformity remains within acceptable limits [22]. These findings suggest that timing should be anchored to documented progression rather than age alone, while remaining sensitive to configuration-specific growth potential and thoracic risk.

For long-segment deformities or extensive MVAs, growth-friendly strategies are commonly described to preserve spinal and thoracic growth, including growing rod constructs, magnetically controlled rods, growth-guidance systems, and VEPTR in selected thoracic insufficiency scenarios [1,12,16,19,34].

### 4.5. Thoracic and Pulmonary Considerations in Treatment Urgency

Thoracic insufficiency syndrome shifts treatment goals beyond radiographic alignment toward preservation of thoracic volume and lung development [5,10,38]. In children with rib fusion or marked thoracic restriction, the literature describes early thoracic expansion strategies (most commonly VEPTR) as an option to support thoracic growth during critical developmental windows [8,20,38]. In contrast, when deformity is primarily lumbar or thoracic volume is relatively preserved, timing may be driven more by curve progression patterns than by pulmonary considerations. Therefore, thoracic and pulmonary evaluation can function as a key modifier of urgency and strategy selection in MVAs, complementing configuration- and growth-based risk assessment.

## 5. Limitations

This review has several limitations inherent to its narrative design. The absence of formal systematic selection and quantitative synthesis may introduce potential selection bias and limits the ability to derive pooled estimates of treatment effect. Furthermore, the heterogeneity of congenital anomaly patterns and variability in reported outcomes across studies restrict direct comparison between treatment strategies. Finally, many available studies remain retrospective and include relatively small cohorts, particularly in patients with multiple vertebral anomalies, which limits the strength of evidence supporting risk stratification models.

## 6. Future Perspectives and Research Directions

Substantial progress has been achieved in understanding and managing congenital scoliosis; however, significant challenges remain, particularly in patients with multiple vertebral anomalies. Future advances are likely to focus on improving risk prediction, individualization of treatment strategies, and preservation of growth while minimizing surgical burden.

Ongoing genetic and molecular research continues to clarify pathways involved in vertebral segmentation and growth, potentially enhancing early risk stratification and targeted surveillance. Although direct biological intervention remains speculative, deeper understanding of developmental signaling pathways may refine prognostic assessment.

Predictive modeling and computational approaches represent an emerging direction for improving clinical decision-making. Integration of anomaly configuration, growth parameters, and radiographic characteristics into predictive frameworks may support more accurate forecasting of curve progression and optimized timing of intervention.

Technological innovation in growth-preserving instrumentation aims to improve durability, reduce complication rates, and decrease the need for repeated procedures. Continued refinement of magnetically controlled systems and growth-guidance constructs may enhance long-term outcomes.

Long-term follow-up studies remain essential as increasing numbers of patients treated with growth-preserving techniques reach skeletal maturity. Robust data on pulmonary function, spinal mobility, and quality of life will be critical for establishing evidence-based treatment algorithms.

Overall, the future of congenital scoliosis management lies in more predictive, biologically informed, and growth-conscious strategies tailored to the complex dynamics of multiple vertebral anomalies.

## 7. Conclusions

Congenital scoliosis associated with multiple vertebral anomalies should be regarded as a distinct biological and mechanical condition rather than merely a more complex form of isolated vertebral malformations. In these patients, deformity progression reflects the cumulative interaction of anomaly configuration, residual segmental growth potential, and mechanobiological amplification during growth, resulting in highly heterogeneous and growth-dependent deformity behavior. Recognition of these dynamics has important clinical implications. Management strategies should move beyond purely angle-based thresholds and incorporate configuration-dependent growth potential, thoracic involvement, and age-related growth velocity when determining surveillance intensity and timing of intervention. Such an approach may allow earlier identification of high-risk deformities while avoiding unnecessary premature fusion in lower-risk configurations. Future prospective studies are required to validate configuration-based progression models and to develop predictive algorithms capable of guiding individualized timing of intervention in this challenging patient population.

## Figures and Tables

**Table 1:** Risk patterns and preferred timing and treatment strategies in congenital scoliosis with multiple vertebral anomalies.

Anomaly Pattern	Growth Potential	Risk of Progression	Typical Clinical Behavior	Preferred Timing of Intervention	Preferred Strategy	Key Supporting References
Fully segmented hemivertebra (isolated or dominant lesion)	High (two active growth plates)	High	Progressive short-segment curve, often early childhood	Early childhood (1–4 years) if progression documented	Hemivertebra resection with short-segment fusion	[1,2,20,21,23]
Multiple fully segmented hemivertebrae on the same side	Very high (additive asymmetric growth)	Very high	Steep, rapidly progressive short-segment curves	Early intervention, usually before 4–5 years	Selective hemivertebra resection ± growth-friendly systems	[1,12,20,23]
Alternating hemivertebrae on opposite sides	Variable (partial growth compensation)	Moderate	Balanced double curves; slower coronal but frequent sagittal progression	Individualized; close surveillance during growth spurts	Observation or selective surgery if progression occurs	[12,20,23]
Unsegmented bar (isolated)	Low or absent on concave side	Moderate	Progressive deformity due to unilateral growth arrest	Early childhood if progression observed	Convex hemiepiphysiodesis or limited fusion	[20,23,25]
Unsegmented bar with contralateral hemivertebra	Extreme asymmetry (growth + arrest)	Very high	Relentless progression, often >5°/year	Very early surgery (often <3–4 years)	Early hemivertebra resection ± hemiepiphysiodesis; growth-friendly constructs for long curves	[1,20,22,23]
Mixed formation and segmentation defects (multilevel)	Heterogeneous	High	Complex 3D deformities, kyphoscoliosis common	Early risk-based intervention	Individualized: selective resection, guided growth, or growth-friendly instrumentation	[1,12,20,23]
Block vertebrae/incarcerated hemivertebrae	Low	Low	Often stable or slowly progressive	Observation	Clinical and radiographic surveillance	[2,20,23]
Thoracic anomalies with rib fusion/thoracic insufficiency syndrome	Variable; thoracic growth restricted	High (functional impact)	Impaired chest growth, pulmonary compromise	Very early (infancy–toddler) if thoracic insufficiency syndrome (TIS) present	Vertical Expandable Prosthetic Titanium Rib (VEPTR) or thoracic expansion techniques	[4,9,34,37]
Long-segment deformity with multiple anomalies	Variable	High	Progressive long curves, limited fusion tolerance	Early growth-preserving phase	Growing rods, magnetically controlled rods, Shilla; delayed final fusion	[1,12,19]

## Data Availability

The original contributions presented in this study are included in the article. Further inquiries can be directed to the corresponding author.

## Abbreviations

The following abbreviations are used in this manuscript:

CS	Congenital scoliosis
CT	computed tomography
VACTERL	A rare, non-random, and typically non-hereditary association of congenital malformations (Vertebral defects, Anal atresia, Cardiac defects, Tracheo-esophageal fistula (TEF), Esophageal atresia, Renal anomalies, and Limb abnormalities)
VEPTR	vertical expandable prosthetic titanium ribs
CVMs	congenital vertebral malformations
TIS	thoracic insufficiency syndrome
